# Growth patterns and associated risk factors of congenital malformations in twins

**DOI:** 10.1186/s13052-020-00838-z

**Published:** 2020-05-24

**Authors:** Ettore Piro, Ingrid Anne Mandy Schierz, Gregorio Serra, Giuseppe Puccio, Mario Giuffrè, Giovanni Corsello

**Affiliations:** Department of Health Promotion Sciences, Maternal and Infant Care, Internal Medicine and Medical Specialties “G. D’Alessandro”, University of Palermo, Piazza delle Cliniche, 2, 90127 Palermo, Italy

**Keywords:** Retrospective study, Multiple birth, Congenital abnormalities, Birthweight discordance, Preterm infant, Microcephaly

## Abstract

**Background:**

The rate of twinning continues to increase due to the combined effect of a rise in parental age and increased use of assisted reproductive technology. The risk of congenital anomalies in twins is higher than in singletons, but it is less well reported in relation to growth patterns. We focused to the auxological outcome of twin pregnancies when one or both of twins are affected by one or more malformations.

**Methods:**

We conducted a retrospective observational study reviewing the clinical charts of twins admitted in the period between January 2003 and December 2018 at the University Hospital of Palermo. The associations between malformations and anthropometric variables at birth were analyzed by comparison within each twin pair and regarding each variable as ordered difference between the two twins.

**Results:**

We studied data of 488 neonates (52% females) from 244 pregnancies. The rate of major congenital anomalies was 11%, affecting significantly the smaller twin (*p =* .00018; Odds ratio 3.21; 95% CI 1.65 6.59). Malformation class distribution was as following: genitourinary (24%), gastrointestinal (20%), cardiovascular (18.5%), musculoskeletal (11%), central nervous system (9%), syndromic (9%), ocular (5.5%) and diaphragmatic hernia (2%). The most predictive value, the Birthweight (BW) difference mean ratio in malformed versus not malformed neonates (− 0.31 vs 0.02; *p =* .0016) was distributed equally lower than zero in all malformed twins, except for those with congenital heart defects (*p =* .0000083).

Microcephaly (head circumference < 2 standard deviation, SD) was present in 3% of symmetrically smaller twin, and severe microcephaly (< 3 SD) was present in 0.6%. We found that an intertwin BW discordance of 18% or greater identified 50% of neonates with microcephaly, but only 11% of malformed twins.

**Conclusions:**

In case of one twin with a BW < 10th centile, a concomitant intertwin BW discordance ≥18% could reveal an increased risk for microcephaly but not for malformation. Lower values of BW, Ponderal index, Body mass index but above all negative value of BW difference mean ratio are associated with malformations in twin pairs.

## Background

In the last years, in relation to modernization of societies and consequent advanced parental age, we assisted to a progressive falling in fertility rate. Actually, in more developed nations, about one in six couples is infertile with a reported prevalence of infertility 12.5% among women and 10.1% among men. Thus, more than half of these subjects need medically assisted reproduction consisting of hormonal treatments and assisted reproductive technology (ART) [1], along with a reported certain reluctance to talking about conceiving their children using medical or professional help [2].

In high-income countries consequently, a concomitant increase of multiple birth has been observed, reaching approximately 3% of live births producing significantly more dizygotic (DZ) twins than would occur spontaneously in nature [3]. Since the first reported case series in Italy over 30 years ago [4], the pregnancy rate by ART in Italy and in Europe has declined from 34 to 17%, while the ART single delivery rate is increasing from 78.6 to 83.5%, ART multiple delivery rate progressively is declining from 21.4 to 18.1% (twins at 17.3%, triplets at 0.8%) and preimplant genetic testing is increasing [5].

Also, spontaneous twinning rates are increased with advanced parental age and urban residence confirming an environmental influence [6].

Irrespective of mode of conception, twins in comparison to singletons, show an increased risk of prenatal death, chromosomal abnormalities, congenital malformations, prematurity, perinatal mortality and morbidity [3–7]. Moreover, fetal growth restriction (FGR); also defined as intrauterine growth restriction, and the condition of small for gestational age (SGA), frequently used interchangeably, are also more frequent in case of multiple pregnancy, with a higher global complication rate affecting monozygotic (MZ) twins [7, 8]. FGR, variably defined in scientific literature, cannot be frequently predicted by maternal risk factors [8], and contribute to the well documented higher prevalence of neurodevelopmental impairment in selected categories of twins compared to singletons [9].

A recent study, conducted on MZ and DZ twin pairs, confirmed that both genetic and environmental factors exert their influence on birth size, and assessed that shared environmental factors are prevalent on genetic ones [9]. During the first and second trimester of multiple pregnancies the fetal growth pattern is similar to that of singletons, while from usually 30 weeks of gestation, in relation to inability of the uterine milieu to equally nurture twins, a progressive reduction in growth velocity happens, with lower birthweight (BW) compared to singletons [10]. Outcome measures of intrauterine growth like BW, a crude measure of fetal growth, or specific biometric indices, as Quetelet’s Body mass index (BMI) or Rohrer’s Ponderal index (PI), have been used by clinicians to better compare body composition and symmetry. Especially lower values of PI characterize a relative asymmetric pattern of intrauterine growth, occurring in the third trimester and consisting of an initial reduction in the fetal weight growth rate with subsequent slowing of length growth rate [11]. An asymmetric growth pattern has been found to be associated to delayed visual pathways myelination in twins [12].

The intra-twin pair difference in BW are greater for monochorionic (MC) twins due to uterine crowding, uteroplacental insufficiency and asymmetric cord insertion [13]. Thus, in the smaller twin a marginal or velamentous cord insertion is present, by contrast a larger twin’s cord centrally or eccentrically inserted is reported in the heavier twin [14].

In literature different values have been found to define the weight discordance between the fetuses. Values equal or greater than 15, 18, 20, 25, 30% or 35% have been used in studies on different outcomes in twins and higher order pregnancies [15–20]. Neonatal mortality rate and some morbidities of the smaller twin significantly increase with increasing discordance from 15 to 30% by 5.6 to 43.4% [21]. Larger twins with discordance greater than 30% were at greater risk for significant morbidities like patent ductus arteriosus, cerebral palsy, retinopathy of prematurity, and neurodevelopmental impairment [12, 21].

Similarly, although the overall risk of congenital malformation in twin is about 6%, in MC twins a two to three-fold higher rate than in dichorionic (DC) twins is reported. Midline defects and congenital heart defects (CHD) are particularly increased in MC twins [22].

Since studies on malformation in twin pregnancies have not focused on specific growth indices able to identify, independently from chorionicity, the affected twin within the couple, we performed a research on twins aiming to define a threshold value of indices related to intertwin growth difference between normal and affected twin within the couple.

## Methods

This retrospective observational study was conducted reviewing the clinical charts of twins admitted in the period between January 2003 and December 2018 at the University Hospital of Palermo. Ethical approval was granted from the Ethics Committee Palermo 1 of the University Hospital. The study population consisted of 488 twins (52% female), born from 244 pregnancies. All newborns included were from Italian parents.

Gestational age (GA) in weeks was calculated from maternal last menstrual date or from date of embryo transfer in ART and confirmed by Dubowitz examination. Zygosity tests were not available.

Neonatal auxological parameters were reviewed, including BW in g, length (L) in cm, head circumference (HC) in cm, and two biometric indices were considered: Ponderal index (PI = birthweight*100/length^3^), and Body Mass Index (BMI = birthweight*10/length^2^). As neonatal centile charts for twins were not available for Italian population, we included in the analysis the results obtained from the adoption of the Italian neonatal anthropometric charts for singletons [23].

We defined twin A and twin B in relation to the higher and lower BW, respectively in each twin pair, and each variable was expressed as the difference between the value in twin A minus the value in twin B. We used differences because they are absolute effect measures.

We analyzed in each couple the following indices: difference in BW (BW_A_-BW_B_), BW discordance [(BW_A_-BW_B_)/BW_A_]_,_ and difference in BW respect to the BW mean of the couple [BW difference mean ratio = BW_A_-BW_B_)/(BW_A_ + BW_B_)/2]. The latter biometrical index expresses not only the intertwin BW discordance but also categorizes the intertwin BW difference in binary form, as positive or negative.

Only major malformations were considered and classified according the International Classification of Diseases, tenth revision (ICD-10) in diagnostic codes Q00-Q99 (congenital malformations, deformations and chromosomal abnormalities). Moderate microcephaly (< 2 standard deviation, SD) and severe microcephaly (< 3 SD) were also recorded (Q02 of ICD-10).

Statistical analyses were performed by the open source statistical R 3.04.0 software (R Development Core Team, Vienna, Austria), using for categorical variables chi-squared tests and Fisher tests and for continuous variables t-tests, Wilcoxon-tests and Kruskal-Wallis-tests. The significance was defined as *p* value <.05. Logistic regressions were used to compare continuous variables. Odds Ratios with 95% confidence intervals (CI) were calculated. Stepdown logistic regression models selecting the most significant variables to predict the variable “malformation” were built and predictive accuracy of the best performing model was set by McFadden’s and Nagelkerke’s pseudo R2.

## Results

### Twin characteristics

We studied data of 488 neonates (52% females) from 244 pregnancies with complete auxological data in the couple. The mean values of considered variables characterizing our population were: GA 35.2 ± 2.9 weeks, preterm rate (< 37 weeks) 65.9%, very preterm rate (< 32 weeks) 12.7%, BW 2146 ± 595 g (females 2103 ± 557 g, males 2192 ± 633 g), low BW rate (< 2500 g) 72%, L 44.3 ± 4.2 cm, HC 31.4 ± 2.7 cm, PI 2.4 ± 0.35 and BMI 10.7 ± 1.7.

FGR considered as BW < 3rd centile occurred in 36/488 (7.4%) of neonates, and in 44/488 (9.0%) of neonates when a BW < 10th centile and concomitant BW discordance of 18% or greater were considered.

Chorionicity, amnionicity and their percent distribution were: DC diamniotic (DCDA) 80.4%, MC diamniotic (MCDA) 17.6% and MC monoamniotic (MCMA) 2%. Sex was concordant in all MCMA and MCDA pregnancies, and in 32.5% of DCDA pregnancies.

The reported prevalence of major malformations was 11% affecting more frequently the smaller twin (40/54; 74%), and in 12/54 (12%) both twins. Odds ratio for malformation in the smaller twin was 3.21 (95% CI 1.65 6.59). In the DCDA-MCDA-MCMA subgroups, the frequency of malformations was respectively distributed as follows: 23/246 (9%) – 6/54 (11%) – 2/6 (33%). Anatomical site and percent distribution of malformations in the whole sample were as follows: genitourinary (13/54; 24%), gastrointestinal (11/54; 20%), cardiovascular (10/54; 18.5%), musculoskeletal (6/54; 11%), CNS (5/54; 9%), syndromic (5/54; 9%), ocular (3/54; 5.5%) and diaphragmatic hernia (1/54; 2%). In case of MC placentation, only CHD, SNC and syndromic malformations were encountered, whereas in the DC group the genitourinary malformation was prevalent. Concordant malformations in four female twin pairs were gastrointestinal (duodenal atresia), cardiovascular (interventricular septum defect) and ocular malformations (congenital glaucoma). Discordant malformations in one sex-discordant twin pair were cardiovascular malformation (atrioventricular canal) in the male and gastrointestinal malformation (malformation of intestinal fixation) in the female counterpart. Two other discordant malformations in male twins, cardiovascular (interventricular septum defect) and gastrointestinal (congenital obstruction of small intestine) malformations, were all associated with genitourinary anomalies (cryptorchidism and hypospadias, respectively).

Microcephaly (HC < 2 SD) was present in 16/488 neonates (3%), all of whom were symmetrically small twin B. Severe microcephaly (HC < 3 SD) was present in 3/488 neonates (0.6%). Of these, anencephaly was found as a discordant malformation in two MCMA twin pairs.

Macrocephaly (HC > 2 SD) was present in 9/488 neonates (1.8%).

### Auxological characteristics of malformed twins

Malformations resulted correlated with some measured auxological parameters (Table 1). Malformed versus non malformed neonates had lower values of BW (1915 vs 2270 g; *p =* .00113), L (43.0 vs 45.2 cm; *p =* .00280), and HC (31.0 vs 32.0 cm; *p =* .01571). GA of couples with one or both twins affected by malformation was not significantly lower (35.7 vs 36.1 weeks; *p =* .05428). Adjusted for GA, there were no relationship between percentiles of BW, being SGA, L, and HC. There were also no differences in potential maternal determinants of growth impairment such as age, parity, maternal medications and pathologies or gestational diabetes mellitus. Except for two cases of anencephaly and another case of diaphragmatic hernia in a context of a proximal 6q deletion syndrome, malformations did not contribute to the neonatal mortality rate of 2% recorded in the whole sample. Mortality rate was only correlated to extreme prematurity (median 24.3 weeks’ gestation, *p =* .00002).

**Table 1:** Demographic and auxological characteristics of twin neonates

	Not malformed neonates (*n* = 434)				Malformed neonates (*n* = 54)				***p***
	Mean	SD	Median	IQR	Mean	SD	Median	IQR	
**Twin A, larger (%)**	94.3				5.7				
**Twin B, smaller (%)**	86.6				16.4				***0.00018***
**Sex (F:M)**	1.0				1.5				*0.26080*
**Prematurity (%)**	65.0				74.0				*0.18330*
**GA (weeks)**	35.3	2.9	36.1	2.9	34.5	3.1	35.7	4.9	*0.05421*
**BW (g)**	2177.5	584.7	2270.0	697.5	1892.2	628.5	1915.0	855.0	***0.00113***
**BW centile**	30.5	24.8	24.0	36.0	27.0	26.0	17.5	35.0	*0.13920*
**SGA (<10° centile, %)**	31.5				54.3				*0.07327*
**FGR (< 3° centile, %)**	7.1				9.3				*0.57470*
**BW difference (g)**	275.4	247.1	200.0	270.0	311.3	281.4	210.0	210.0	*0.36720*
**BW discordance**	0.11	0.10	0.09	0.11	0.15	0.13	0.12	0.10	*0.10930*
**BW difference mean rate**	0.01	0.09	0.01	0.09	−0.05	0.11	−0.05	0.09	***< 0.0001***
**Length (cm)**	44.5	4.2	45.2	4.0	42.8	4.5	43.0	5.0	***0.00280***
**Length centile**	34.5	27.4	29.0	44.0	31.3	27.4	27.0	43.8	*0.30510*
**Length difference (cm)**	1.4	1.9	1.0	2.5	1.7	2.8	1.0	3.0	*0.99490*
**HC (cm)**	31.5	2.8	32.0	2.5	30.5	3.1	31.0	4.6	***0.01571***
**HC centile**	41.1	27.4	36.0	40.4	40.4	30.3	35.5	50.8	*0.66650*
**HC SD**	−0.27	0.97	−0.35	1.18	−0.44	1.24	−0.38	1.43	*0.61490*
**HC difference (cm)**	0.69	1.30	0.50	1.40	0.82	1.79	0.70	1.50	*0.66060*
**PI (g/cm**^**3**^**)**	2.4	0.3	2.4	0.3	2.3	0.3	2.3	0.4	***0.02298***
**PI difference**	0.08	0.29	0.05	0.37	0.10	0.30	0.13	0.33	*0.2140*
**BMI (g/cm**^**2**^**)**	10.8	1.6	11.0	1.8	10.0	1.8	9.9	2.8	***0.00207***
**BMI difference**	0.70	1.09	0.54	1.37	0.87	1.10	0.76	1.33	*0.08006*
**BMI difference mean ratio**	0.03	1.14	0.02	0.70	−0.25	0.65	−0.31	0.87	***0.00159***

*Abbreviations*: *BMI* body mass index, *BW* birthweight, *FGR* fetal growth restriction, *GA* gestational age, *HC* head circumference, *PI* ponderal index, *SD* standard deviation, *SGA* small for gestational age

*Significant differences are in bold*

**Fig. 1:**
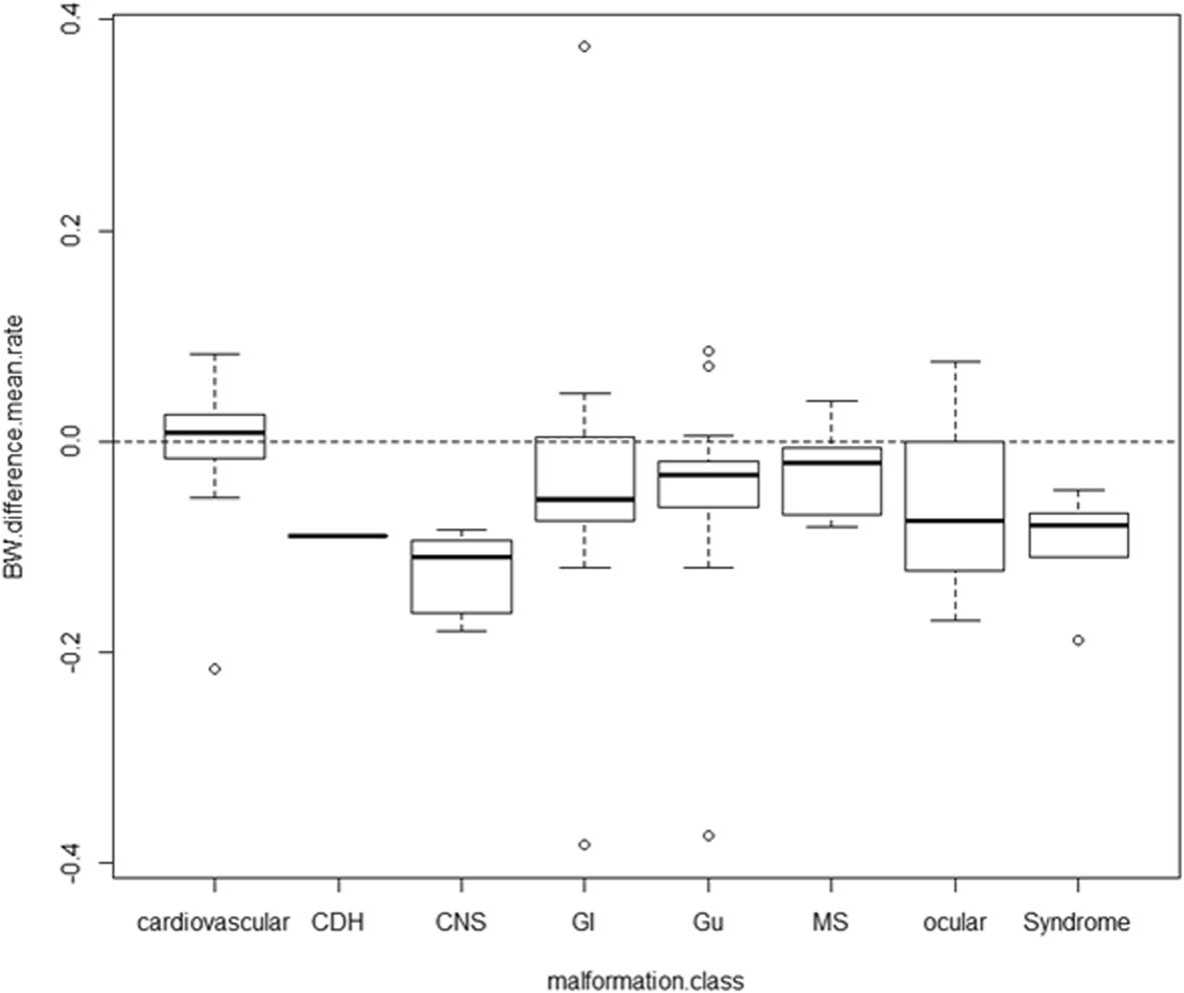
Malformation metrics to intertwin birthweight difference mean ratio: Malformation class distribution are reported as box plots and are distributed equally lower than zero in all malformed twins, except for those with cardiovascular malformations (*p =* .0000083). Solid circles in the box plot graphs represent outliers with values that lies more *than* one and a half *times* the length of the box. BW, birthweight; CDH, congenital diaphragmatic hernia; CNS, central nervous system GI, gastrointestinal; Gu, genitourinary; MS, musculoskeletal malformation

The most used anthropometric indices were significantly lower in twins affected by malformations, BMI (9.9 vs 11.0; *p =* .00207) and PI (2.3 vs 2.4; *p =* .02298). Among the proposed anthropometric combined indices the followings resulted significant: BW difference mean ratio (− 0.31 vs 0.02; *p =* .00159), BMI difference mean ratio (− 0.0498 vs 0.0062; *p =* .000013), and PI difference mean ratio (− 0.05 vs 0.01; *p =* .04931). All these were distributed equally lower than zero in all malformed twins, except for those with cardiovascular malformations (Fig. 1).

FGR considered as BW < 3rd centile occurred in 36/488 neonates, 5 of which had malformations (14%; *p =* .5747) independently of their sex; the same observation was found considering a BW < 10th centile and concomitant discordance of 18% or greater (44/488 neonates), present in 5 malformed neonates (11%; *p =* .94730). No newborn with cardiovascular malformation presented microcephaly and there were no differences in HC SDs (*Z*-scores) between this malformation, other malformations and non-malformed counterparts (− 0.620 vs − 0.215 vs − 0.350; *p =* .8743).

We found that an intertwin BW discordance of 18% or greater identified 8/16 (50%) of neonates with microcephaly.

Stepdown regression analysis evidenced that a lower crude BW (*p =* .04802) associated to BW difference mean ratio (*p* = .00276) were the most predictive determinants for malformations. Significance of this model (Nagelkerke’s method) was very high (*p =* .0000426), with a calculated accuracy of 74.4%.

## Discussion

This study investigated the relationship between congenital malformations and intrauterine growth patterns assessed at birth in twin pairs.

### Twin characteristics

There were similarities in twin characteristics in our population respect to previous studies, as high frequencies of very preterm (12.7% vs 13.6%), preterm (65.9% vs 41.7–59.9%) and very low BW neonates (13.9% vs 9.5%) [5, 24], but there were differences in reported rate of congenital malformations in twinning (11%). This higher prevalence than previously reported (2–9%) [20, 25–27], is probably related to the presence in our institution of an obstetrical and surgical reference center for western Sicily. However, the study can be considered representative [20, 25–27], because stochastics of factor of chorionicity and type of malformations are normally distributed [28].

Previous studies have suggested that a discordant malformation in MC twins represents one of the more important clinical problems affecting a significant proportion of perinatal morbidity and mortality [16], and that the magnitude of the association between twinning and malformations varies by zygosity, with stronger association reported for MZ twins than for DZ twins except for a few particular birth defects (anotia/microtia, transposition of great arteries, CDH and craniosynostosis) [29]. Twinning can represent also a significant medical risk for maternal complications such as gestational diabetes mellitus, pregnancy induced hypertension, anemia or preeclampsia, leading to neonatal complications as FGR, low BW, preterm birth, mortality, or malformations [3, 19, 29].

The intent of the study was to analyze auxological patterns in malformed twin pairs. There were no differences in sex or gestational age.

### Auxological characteristics of malformed twins

In the scientific literature chorionicity, neonatal weight and the birthweight difference were the most studied parameters to analyze neonatal mortality, and morbidity in multiple pregnancies. Neonatal death among the severely discordant smaller twin (≥35%) is significantly more frequent than in a non-discordant smaller twin with a greater effect in case of concomitant condition of SGA [15]. Such a severe birthweight discordance was 2.5-fold higher in triplets [20]. In another study a birthweight discordance exceeding 18% was associated with a minimum twofold increase in risk of perinatal morbidity both for dichorionic and monochorionic twin pairs without twin-twin transfusion syndrome, and persisting even when both twins were adequate to GA [17]. In another study a birthweight discordance ≥30% has been associated with low Apgar score and perinatal mortality [19].

It is known that a smaller fetus has a higher risk to have a chromosomal disorder or another congenital malformation, but to our knowledge there are still no report that constates that the smaller fetus in twin pairs is the more likely affected by malformations. In our sample the absence of *hydrops fetalis* excluded an increased false positivity of auxological indices.

We found that the malformed twin was smaller, shorter, with lower HC, lower BMI and lower PI. As seen in a previous study, the mean BW difference and BW discordance were not significant augmented [30]. Thus, aiming to find a new growth-related parameter or index that could predict the occurrence of congenital malformations in twins, we identified the BW difference mean ratio. This new index resulted to have the most important predictive value for all categories of malformations except for CHD. BW difference mean ratio was not subdued by influence of sex.

The commonly used criterion of FGR ascertained only 11–14% of malformed twins. Indices related to length which are independent of sex in fetuses or neonates in our population correlated with malformations.

Moderate microcephaly occurred a hundred times more frequent in twins (1/30) than in the general population, also, severe microcephaly occurred more frequently (1/163) [28].

We think that the distribution of the HC values in the whole twin sample is shifted to the left if compared to normal values. Indeed, we found a HC <2SD, accounting for 3% of subjects, a slightly higher value than expected (2.3%). All of these twins where symmetrically small and with a lower intertwin birthweight, thus, we suppose, without a fetal adaptive mechanism of brain sparing phenomenon. A HD < 3SD has been found in 0.6% of subjects. Thus, severe microcephaly was more frequent than expected (0.13%), and we think related to a higher incidence of genetic and malformative pathologies affecting the CNS in our twin sample.

A HC >2SD was present in 1.8% of twins, a lightly lower value than expected (2.3%), not related to the symmetricity of the growth pattern or higher intertwin birthweight.

Despite the overall concerning neurodevelopmental deficits in children with CHD needing surgery or not and the high incidence of microcephaly at birth in singletons with several forms of CDH [31], we confirm the findings from Schultz and colleagues that in affected twins are no differences in HC respect to their counterparts [32].

Indeed, neonates with CHD showed a better fetal growth than neonates with other classes of malformation because of the preserved placental circulation and the likely spontaneous fetal demise of complex or severe CHD. The latter fact as well as our retrospective study design could be an explanation for the finding that prevalence of CHD was lower than expected (2% vs 6%) [33]. In this previous study, in which more ART patients were recruited, monochorionicity was a determinant of CHD [33], as observed by us. In MZ twins, discordant malformations, especially with malformations of the cardiovascular and digestive system are reported, and epigenetic and environmental factors would prevail on genetic post-twinning de novo alterations [34–36].

Since twins are also at risk of acquired cardiac dysfunction, occurring more frequently in MZ twins as a result of twin-to-twin transfusion syndrome or placental shunts [37], we propose to perform a fetal and neonatal echocardiography to exclude both structural and functional anomalies. Recently it was confirmed that the larger twin is at risk to develop cardiac organomegaly and insufficiency also in absence of twin-to-twin transfusion syndrome [38]. Some malformations of various organ systems could be recognizable only beyond the neonatal age and suspected only if neurodevelopment is not adequate for postnatal age [39]. Therefore, it is justified to carry out a targeted planning of the neurodevelopmental and behavioral assessment in all twin-pairs [40].

In the evaluation of FGR we combined the 10th centile normally used in singletons with an intertwin BW discordance of 18% or greater. Adopting this associated index, we have selected 9% of the total number of twins in our sample, a value coinciding with a value inferior to the 10th centile, universally considered as the threshold for the definition of the SGA newborn. This new combined index (value of BW < 10th centile and concomitant BW discordance ≥18%), could therefore be proposed as a threshold value for the definition of the SGA twin within the couple.

## Conclusions

In case of one twin with a BW <10th centile, a concomitant intertwin BW discordance ≥18% could reveal an increased risk for microcephaly but not for malformation. Despite lower values of BW, PI and BMI are associated to malformations in twin pairs, we conclude that the best predictive index for fetal assessment is a negative value of BW difference mean ratio. CHD are the unique type of malformation not in accordance with these findings. Thus, a fetal and neonatal echocardiography should be performed in all twins to exclude structural and functional cardiovascular anomalies.

## Data Availability

The datasets used and/or analyzed during the current study are available from the corresponding author on reasonable request.

## Abbreviations

ART: Assisted reproductive technology
BMI: Body mass index
BW: Birthweight
CDH: Congenital diaphragmatic hernia
CHD: Congenital heart defects
DC: Dichorionic
DCDA: Dichorionic diamniotic
DZ: Dizygotic
FGR: Fetal growth restriction
GA: Gestational age
HC: Head circumference
ICD-10: International Classification of Diseases tenth revision
L: Length
MC: Monochorionic
MCDA: Monochorionic diamniotic
MCMA: Monochorionic monoamniotic
MZ: Monozygotic
SD: Standard deviation
SGA: Small for gestational age
US: Ultrasound
VSD: Ventricular septal defects
